# The Prevalence of HIV Seroconversion in Healthcare Workers Following Sharp Injuries and Exposure to Biofluids

**DOI:** 10.7759/cureus.66773

**Published:** 2024-08-13

**Authors:** Jorge Luis Pineda-Ramirez, Erick Sierra-Diaz, Eugenio Vladimir Zavala-Sánchez, Guadalupe Zarate-Leal, Diana Lorena Cisneros-García, Eduardo Alfonso Hernández-Muñoz, Jose de Jesus Guerrero-García, Adrian Ramirez-De Arellano

**Affiliations:** 1 Preventive Medicine, Instituto Mexicano del Seguro Social, Guadalajara, MEX; 2 Epidemiology and Public Health, Instituto Mexicano del Seguro Social, Guadalajara, MEX; 3 Epidemiology, Instituto Mexicano del Seguro Social, Guadalajara, MEX; 4 Public Health, Universidad de Guadalajara, Guadalajara, MEX; 5 Central Blood Bank, Instituto Mexicano del Seguro Social, Guadalajara, MEX; 6 Biomedical Science Laboratory, Universidad de Guadalajara, Guadalajara, MEX

**Keywords:** workplace accidents, healthcare workers, seroconversion, biofluids exposure, sharp injuries, human immunodeficiency virus

## Abstract

Background and objective

Workplace accidents (WPAs) are a common problem worldwide. They are often considered a public health concern due to the potential transmission of infections such as HIV, hepatitis B, and hepatitis C through sharp devices or direct exposure to biofluids. Post-exposure prophylaxis (PEP) has demonstrated effectiveness in such instances, especially immediately after exposure. The present study aimed to report the prevalence rate of HIV seroconversion following such exposure among healthcare workers (HCWs).

Methods

We conducted a cross-sectional study involving a database analysis of cases from 2015 to 2024. Central tendency measures were used to describe population characteristics, and rates were calculated using standard methods.

Results

A total of 514 HCWs were included in the study. The prevalence of WPAs was 13 per 100 HCWs. Regarding WPAs related to HIV exposure, the prevalence was 0.9 per 100 HCWs, with 0% seroconversion thanks to timely PEP.

Conclusions

WPAs related to HIV exposure are a serious issue for public health systems worldwide. Although protocols are available and no seroconversion cases were reported in the present study, PEP is not always accessible in several settings, increasing the risk of seroconversion. International public policy measures should be uniformly implemented to provide faster access to prophylaxis, educate the personnel, raise awareness about bloodborne diseases, and reduce excessive red tape.

## Introduction

Healthcare accidents constitute a common public health concern worldwide. As per WHO data released in 2022, more than two million healthcare workers (HCWs) suffered a workplace accident (WPA) [1]. Sharp injuries (SIs) and biofluid exposures remain the most common occupational accidents in medical facilities. According to the Centers for Disease Control and Prevention (CDC), approximately 400,000 sharp-related injuries are reported yearly. The CDC defines a sharp injury as “a penetrating stab wound from a needle, scalpel, or other sharp object that may result in exposure to blood or other body fluids” [2]. Bloodborne infectious diseases are a major concern since they can be life-threatening after transmission [3]. Viral bloodborne infectious diseases such as hepatitis B and C are frequently related to SI in HCWs. In 2005, the WHO reported 66,000 hepatitis B and 16,000 hepatitis C infections related to these injuries [2,4].

In 2018, Kanwugu et al. reported that around 38 million people were living with HIV infection worldwide. More than half of the cases were in Africa, while only 2.2 million lived in Europe and North America [5]. HIV cannot survive outside the bloodstream or lymphatic tissue. The virus must be transmitted through direct biofluids and contaminated sharp materials or medical devices to infect an individual. The infectiousness depends on the virus type, viral load in the biofluids, and host susceptibility. The risk of seroconversion after an accident with a sharp device contaminated with HIV biofluids is around 0.3% [6,7]. In a recent online survey conducted among 460 HCWs in the United States, 59% (n=263) reported experiencing SI; however, only approximately half of them (n=143) involved WPAs [8]. However, other authors have stated that 25-90% of WPAs remain unreported [9].

Nowadays, the natural history of HIV infection is well-understood, and its study constitutes a dynamic aspect of public health science. Nevertheless, HCWs' apprehension about contracting the virus remains a significant concern that needs to be addressed by health systems worldwide. As per the Occupational Safety and Health Administration (OSHA), WPAs have a significant impact on workers' quality of life and they lead to financial challenges for healthcare institutions [10,11]. The present research aims to report the prevalence rate of seroconversion following HIV exposure through sharp devices and contact with biofluids among HCWs.

## Materials and methods

A cross-sectional study was performed using the database of the Department for Prevention and Health Promotion at the Western National Medical Center after obtaining approval from institutional authorities (R-2024-1301-092/COFEPRIS 17 CI 14 039 114/CONBIOETICA 14 CEI 2019 01 23). This medical facility is one of the biggest at the national level and caters to 17,000,000 users in the west of Mexico. The information in the database included HCW accidents from 2015 to 2024. The sampling method used was non-probabilistic and convenience-based, with no sample size calculation performed. The eligibility criteria for the study included the entire population of cases who sought medical attention (n=514) after a work-related sharp injury or biofluids exposure. Before data analysis, any variables that could potentially identify the HCWs were removed; instead, cases were labeled with a consecutive number for internal identification.

Descriptive analysis was performed using central tendency measures, frequencies, and percentages. Prevalence was calculated using the number of WPAs over the total number of HCWs in the medical facility per time period per 1000s. No inferential statistics were used in the present research. Data was analyzed using Excel 365® (Microsoft Corporation, Redmond, WA) and OpenEpi (Open-Source Epidemiologic Statistics for Public Health, Bill and Melinda Gates Foundation, Emory University, Atlanta, GA).

## Results

A total of 514 HCWs were included in the study, all of whom reported a previous history of SI or biofluid exposure in the workplace. Of these, 58% were females (n=299), while the remaining participants were males. The mean age of the cohort was 34.4 years (SD: 7.53, 95% CI: 33.7-35.0). The mean age among females was 34.5 years (SD: 7.3, 95% CI: 33.6-35.3), while it was 34.2 years among males (SD: 7.7, 95% CI: 33.1-35.2). The difference in the mean was calculated using a parametric test (t-test), yielding a value for equal variance of 0.3 (-1.01-1.6) with a p-value for equality of variance >0.05.

Medical devices are constantly in contact with patients and HCWs, posing the risk of WPA and transmission of bloodborne infections. Among the various types of WPA, SIs were the most common, followed by biofluid exposure. Further details are presented in Table 1.

**Table 1:** Sharp injuries and biofluid exposure incidence among healthcare workers by category

Healthcare worker category and accident type	Number of accidents (n=514)	Accidents (%)
Residents	178	34.8%
Sharp injury	139	27.3%
Biofluid exposure	39	7.5%
Nurses	155	30.3%
Sharp injury	140	27.2%
Biofluid exposure	15	3.1%
Maintenance and general services	83	16.1%
Sharp injury	81	15.7%
Biofluid exposure	2	0.4%
Chemists and laboratorists	44	8.5%
Sharp injury	43	8.3%
Biofluid exposure	1	0.2%
Attending physicians	43	8.2%
Sharp injury	38	7.3%
Biofluid exposure	5	0.9%
Technicians	6	1.0%
Sharp injury	3	0.5%
Biofluid exposure	3	0.5%
Other personnel	4	0.8%
Sharp injury	4	0.8%
No data (ND)	1	0.2%
Sharp injury	1	0.2%
Total	514	100%

As can be seen, residents were the most affected HCW category regarding accidents, accounting for 34.8% of accidents (n=178). Among the observed cases, about 7% of HCWs experienced an accident related to HIV exposure, with SI being the most common among residents. Table 2 displays the categories and percentages of WPAs related to HIV exposure.

**Table 2:** Sharp injuries and biofluid exposure incidence related to HIV among healthcare workers by category

Healthcare worker category and accident type	Number of healthcare workers with exposure related to HIV (n=36)	Healthcare workers with exposure related to HIV (%)
Resident	21	58.3%
Sharp injury	12	33.3%
Biofluid exposure	9	25.0%
Nurses	11	30.6%
Sharp injury	9	25.0%
Biofluid exposure	2	5.6%
Attending physicians	3	8.3%
Sharp injury	3	8.3%
Other personnel	1	2.8%
Sharp injury	1	2.8%
Total	36	100%

Post-exposure prophylaxis (PEP) was implemented as per the recommendation by the WHO and the International Labour Organization (ILO) in 2007. Initially, the recommended regimen consisted of zidovudine and lamivudine [12]. However, the currently recommended regimen consists of bictegravir, emtricitabine, and tenofovir [13,14]. Throughout the nine-year study period, three different regimens were used in HCWs exposed to HIV-contaminated medical devices or biofluids (Table 3).

**Table 3:** Post-exposure prophylactic treatment types and schemes applied to healthcare workers by gender

Prophylactic treatment type	Treatment scheme	Female	Male	Total by gender	Seroconversion (%)
Single dosage	Bictegravir + emtricitabine + tenofovir	11	11	22	0%
Complete prophylactic treatment	Emtricitabine + tenofovir and lopinavir + titonavir	2	8	10	0%
	Emtricitabine + tenofovir	3	1	4	0%
	Bictegravir + emtricitabine + tenofovir	8	14	22	0%
Untreated	N/A	276	180	456	N/A
Total		n=300	n=214	n=514	0%

As shown in Table 3, none of the HCWs exposed to HIV through sharp injuries or direct contact with biofluids developed seroconversion during the one-year follow-up period (five tests). Based on the results, the percentage of seroconversion after HIV exposure is zero if PEP is administered promptly and per international guidelines. The overall prevalence of WPAs was 13.4 per 100 HCWs. For HCWs exposed to HIV through SI or biofluids, the prevalence was 0.9 per 100 HCWs.

As mentioned, the total number of WPAs was 514. The most common medical device associated with SI were hypodermic needles, accounting for 56% (n=292). Regarding biofluid exposure, blood was the most common medium, with 7.6% of cases (n=39). Table 4 provides further details on other medical devices associated with WPAs.

**Table 4:** Injuries among healthcare workers by type of medical supply and biofluids

Type of medical supply and biofluids	Number of injuries (n=514)	Injuries (%)
Hypodermic needle	292	56.8%
Lancet	49	9.5%
Blood	39	7.6%
Suture needle	31	6.0%
Scalpel blade	28	5.4%
Biofluid not specified	16	3.1%
Medical knife	16	3.1%
Glass medical supply	14	2.8%
Other medical devices	14	2.8%
Biopsy needle	6	1.2%
Other biofluids	6	1.2%
Other needles	3	0.6%
Total	514	100%

**Figure 1:**
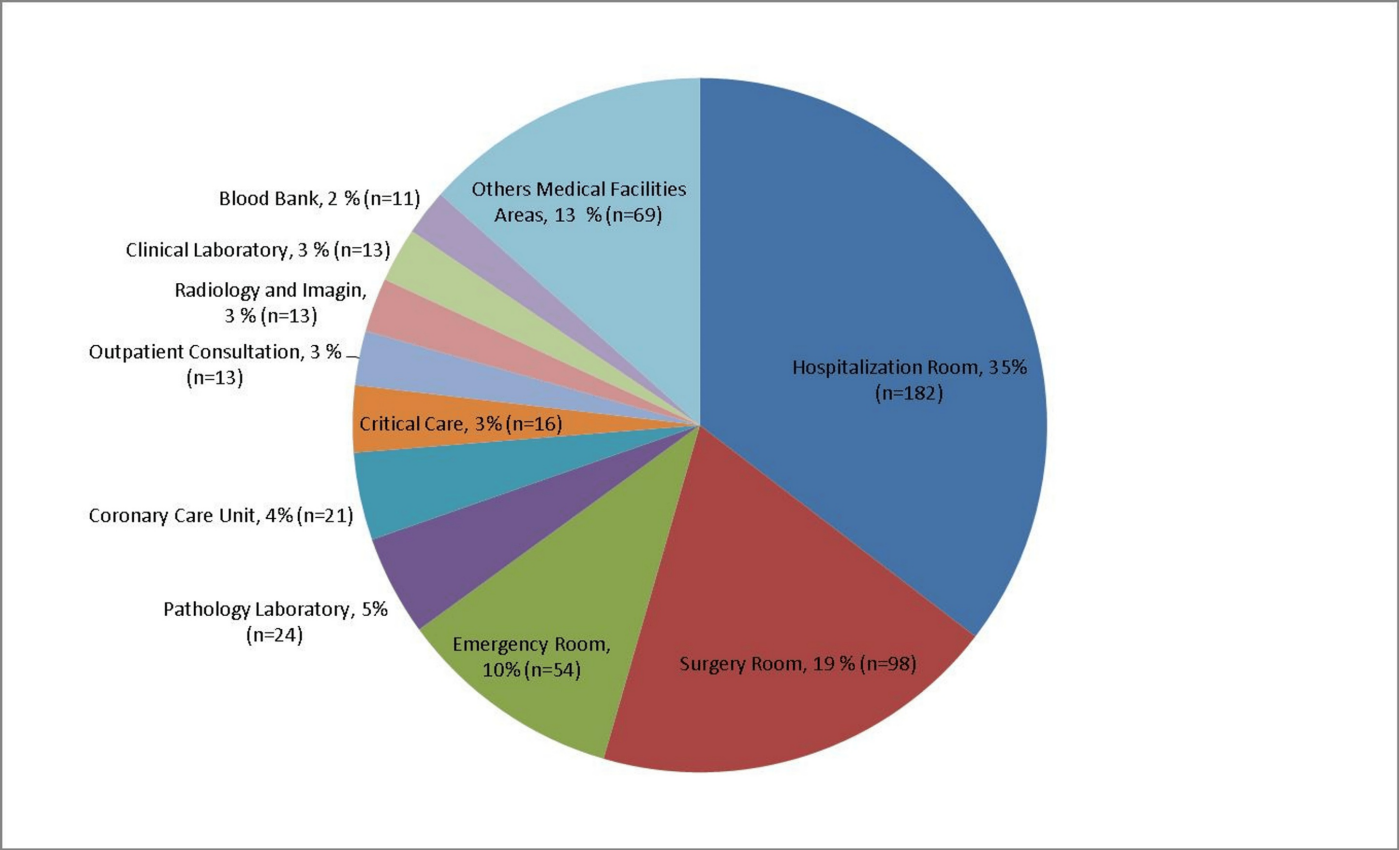
Workplace accidents in different areas of the medical facility

A medical facility is a field ground with several factors posing risks for WPAs. It might be expected that surgery rooms should have the highest frequency of accidents; the present study found that hospitalization rooms were the most common location for accidents, accounting for 35.4% (n=182) of cases, followed by surgery rooms with 19% (n=98). Figure 1 illustrates the details of accidents occurring in various areas within the medical facility.

## Discussion

Viral bloodborne infections are a major concern among HCWs. Seroconversion in HCWs not only diminishes the quality of life but also leads to workplace absences and high costs for both public and private health services. Hence, prevention through effective implementation of PEP protocols is paramount [10,11]. As evidenced in the results section, PEP was successful in preventing seroconversion in 100% of cases. Several factors contribute to the effectiveness of a PEP program [13,14]. Timely notification is the inflection point. On the other hand, health systems must acknowledge the existence of dark numbers, which have the potential to underestimate infection rates. Other aspects of successful PEP implementation include the availability of medication in every healthcare facility, prompt initiation of treatment, a well-structured follow-up plan, and adherence to treatment regimens. Unfortunately, the absence of any of these components leaves gaps that increase the risk of seroconversion.

The medical facility where this research was conducted adheres to federal and international protocols regarding PEP. According to the CDC, HCWs should "Report the exposure to the appropriate person at work and seek medical attention immediately; PEP must be started within 72 hours after an exposure, and careful practice of standard precautions can help reduce exposure while caring for patients with HIV” [15]. The reported events in the current study reflect only the exposures that were notified to the Department for Prevention and Health Promotion. As previously mentioned, more than 50% of workplace exposures are not notified [8].

The concept of "dark numbers" encompasses several contributing factors. One significant factor is the excessive administrative burden related to the event notification that discourages HCWs from adhering to protocols despite being aware of the associated risks. Red tape, characterized by excessive bureaucracy, further complicates decision-making processes and is perceived by workers as a burden. Research on this topic indicates that lower levels of red tape are associated with equity, effectiveness, and better-informed personnel [16,17]. Well-informed HCWs are cognizant of the risks involved, which enhances their performance in self-care activities within a hazardous work environment.

A meta-analysis by Hosseinipalangi et al. (2022) reported that WPAs related to SI are more common in developing countries [1]. The study, based on WHO data, found that the highest incidence rate was observed in the African region, while the lowest rates were reported in Oceania. HIV ranked as the third most common bloodborne disease associated with SI, accounting for 17% of cases, following hepatitis C and B with 21% and 18%, respectively. Hospital rooms were identified as the most common location for WPA, followed by surgery rooms [1]. These findings align with our results. Notwithstanding, some differences should be pointed out. The WHO reported that nurses experienced the highest incidence of WPA, accounting for 56.2% of cases, followed by physicians at 20.2%. In contrast, our research found an inverse result, with physicians accounting for 43% and nurses for 30.1% of WPAs. Nonetheless, similar results were observed in both studies regarding the medical devices associated with SI. As reported by Hosseinipalangi et al., needles were the most common medical tool associated with SI, which agrees with our research as well [1].

In general, WPAs are a common occurrence in medical facilities worldwide. Based on a definition coined by Rittel and Webber in 1976, WPA could become a “wicked problem”. The term implies a problem for which “no solution is found” [18]. The authors stated that despite the availability of numerous courses, training programs, practice sessions, and abundant online resources, the main challenge lies in the lack of interest and motivation among HCWs. Health systems often address only the immediate aftermath of WPA incidents, focusing on the HCWs' response after exposure rather than implementing preventive measures. However, addressing the problem in this limited manner may result in ineffective strategies without significant improvements [18]. The objective should not solely be to reduce the seroconversion rate but to tackle the root cause of WPAs. The most cost-effective and straightforward strategy is to raise awareness through education and foster a culture of prevention. By emphasizing the importance of preventive measures and instilling a proactive mindset among HCWs, the overall number of WPAs can be significantly reduced.

The present study has some important limitations. Since no HCWs developed seroconversion, inferential statistics could not be performed. However, we highlight several issues like the lack of PEP in medical facilities and several cases of WPAs not being notified. Another limitation was the number of notified cases during the first two years. It should be mentioned that in 2015 and 2016 a capacitation program started in the medical facility regarding WPAs. The prevalence of WPAs is depicted in Table 5.

**Table 5:** Prevalence of workplace accidents in the medical facility by year

Year	Prevalence per 100 healthcare workers
2015	0.03
2016	0.25
2017	1.64
2018	2.14
2019	2.58
2020	1.64
2021	1.86
2022	1.86
2023	1.67
2024	0.61

## Conclusions

Our study highlights certain negative externalities caused by a lack of interest on the part of the authorities. Issues such as dark numbers, ignorance, poor protocols, lack of information, apathy among HCWs and the authorities, and the proportion of seroconversion for every bloodborne disease portend serious problems down the line, which could lead to a snowball effect. There is no simple or unique solution to this crisis, with each health system having specific internal issues to tackle. However, implementing preventive measures and raising awareness among HCWs can contribute immensely to addressing the problem.
